# Novel Immune Checkpoint Inhibitor Targets in Advanced or Metastatic Renal Cell Carcinoma: State of the Art and Future Perspectives

**DOI:** 10.3390/jcm13195738

**Published:** 2024-09-26

**Authors:** Samuele Compagno, Chiara Casadio, Linda Galvani, Matteo Rosellini, Andrea Marchetti, Elisa Tassinari, Pietro Piazza, Angelo Mottaran, Matteo Santoni, Riccardo Schiavina, Francesco Massari, Veronica Mollica

**Affiliations:** 1 Medical Oncology, IRCCS, Azienda Ospedaliero-Universitaria di Bologna, 40138 Bologna, Italy; samuele.compagno@studio.unibo.it (S.C.); chiara.casadio9@studio.unibo.it (C.C.); lindagalvani94@gmail.com (L.G.); matteorosellini92@gmail.com (M.R.); andrea.marchetti12@studio.unibo.it (A.M.); elisa.tassinari95@gmail.com (E.T.); francesco.massari@aosp.bo.it (F.M.); 2 Department of Medical and Surgical Sciences (DIMEC), University of Bologna, 40138 Bologna, Italy; pietro.piazza2@unibo.it (P.P.); angelo.mottaran@studio.unibo.it (A.M.); riccardo.schiavina3@unibo.it (R.S.); 3 Division of Urology, IRCCS, Azienda Ospedaliero-Universitaria di Bologna, 40138 Bologna, Italy; 4 Oncology Unit, Macerata Hospital, 62100 Macerata, Italy; mattymo@alice.it

**Keywords:** renal cell carcinoma, RCC, immunotherapy, immune checkpoint inhibitors, ICI, TIGIT, ILT4, LAG-3, TIM-3, vaccine

## Abstract

Immune checkpoint inhibitors (ICI) have become the cornerstone of treatment in renal cell carcinoma (RCC), for both metastatic disease and in an adjuvant setting. However, an adaptive resistance from cancer cells may arise during ICI treatment, therefore many studies are focusing on additional immune checkpoint inhibitor pathways. Promising targets of immunotherapeutic agents under investigation include T cell immunoglobulin and ITIM domain (TIGIT), immunoglobulin-like transcript 4 (ILT4), lymphocyte activation gene-3 (LAG-3), vaccines, T cell immunoglobulin and mucin domain-containing protein 3 (TIM-3), and chimeric antigen receptor (CAR) T cells. In this review of the literature, we recollect the current knowledge of the novel treatment strategies in the field of immunotherapy that are being investigated in RCC and analyze their mechanism of action, their activity and the clinical studies that are currently underway.

## 1. Introduction

In recent years, there has been a progressive increase in the incidence of renal cell carcinoma (RCC), which is associated with a five-year survival rate that has increased from 50% in 1975 to 77% in 2019 [1]. Worldwide, there are over 400,000 new cases of RCC and over 170,000 deaths annually [2].

RCC is divided into two main subtypes, with different clinical and prognostic characteristics: clear cell RCC (ccRCC), which represents the most common histology (75–80% of all cases) and non-clear cell RCC (nccRCC), accounting for 25–20% of RCC and composed of several different histologies, the most common being papillary (15%) and chromophobe (3–5%) RCC [3].

Currently, prognostic factors based on the evaluation of clinical and laboratory values are used to stratify patients with metastatic RCC and therefore define the best therapeutic strategy [4]. However, no validated biomarkers are yet available as predictors of efficacy for current first-line therapies in metastatic RCC (mRCC) [5]. Nowadays, the most used prognostic score for mRCC is the International Metastatic RCC Database Consortium (IMDC) risk score, based on clinical and laboratory criteria, and which stratifies patients into three prognostic groups (favorable, intermediate, and poor risk), each with a different median overall survival (mOS) [6].

In recent years, immune checkpoint inhibitors (ICI) have become the cornerstone of RCC systemic treatment in both metastatic disease and adjuvant settings.

Immuno-based combinations, consisting of an ICI in combination with a tyrosine kinase inhibitor (TKI) or with another ICI, have yielded unprecedented results in several phase III trials conducted on treatment-naive patients with metastatic disease [7].

In the phase III Checkmate-214 trial, the first-line immunotherapy combination of nivolumab (3 mg per kilogram of body weight) plus ipilimumab (1 mg per kilogram) every 3 weeks for four doses, followed by nivolumab (3 mg per kilogram) every 2 weeks, demonstrated a greater overall survival (OS) benefit than 50 mg sunitinib delivered orally once daily in patients with intermediate/poor risk advanced ccRCC [8].

Keynote-426 is an open-label, phase III randomized trial, that demonstrates superiority in terms of OS and progression-free survival for advanced ccRCC (PFS) (co-primary endpoints) of first-line pembrolizumab 200 mg delivered intravenously (IV) every 3 weeks, in combination with 5 mg axitinib delivered orally twice daily, when compared with sunitinib [9].

The CheckMate 9ER phase III study confirmed the efficacy, in terms of PFS (primary endpoint), OS and objective response rate (ORR)—secondary endpoints—of the combination of nivolumab 240 mg IV every 2 weeks and cabozantinib 40 mg orally once daily, versus sunitinib [10].

Lastly, the phase III Clear study demonstrated an advantage in OS and PFS for the use of 20 mg lenvatinib delivered orally once daily in combination with 200 mg pembrolizumab IV every 3 weeks, when compared with sunitinib in advanced ccRCC [11].

The outcomes of first-line immune-based combination phase III trials with a positive OS in mRCC are reported in Table 1.

The outstanding outcomes of these studies led to the approval of the ICI–TKI combination for all IMDC risk class patients or the ICI–ICI combination for the intermediate/poor risk class patients as the standards of care for patients with previously untreated mRCC.

However, adaptive resistance from cancer cells may arise during ICI treatment. Most important is the loss of response to IFNγ and the secretion of immunosuppressive cytokines like IL-6, IL-12 or TGFβ. These cytokines upregulate PD-L1 and create a T cell dysfunction [12]. Moreover, loss of neoantigen expressions leading to evasion from cytotoxic T cell attack have been described in patients with non-small cell lung cancer (NSCLC) [13]. Further potential mechanisms of resistance to ICIs consist of the overexpression of oncogenes and in the gathering of immunosuppressive cell populations inside the tumor microenvironment (TME), such as tumor-associated macrophages (TAM), myeloid-derived suppressor cells (MDSC), and regulatory T cells (Treg). These last two, in particular, seem to be increased in tumor patients and to inhibit T cells activation and function [14,15].

Similarly, resistance to anti-angiogenic therapy may arise specifically due to the increased production of pro-angiogenic factors, endothelial cell variability and cancer cell crosstalk in the tumor microenvironment [16].

After progression on ICI-based first-line treatment, cabozantinib is the most widely used TKI, demonstrating efficacy and safety in several studies as a second-line therapy [17,18]; this is also the case for patients treated with first-line ICI or primary refractory TKI [19].

As an alternative to the use of cabozantinib as a second-line treatment, there are phase II trials and retrospective evidence that show that the combination of lenvatinib plus everolimus leads to a benefit in terms of PFS with an acceptable toxicity profile [20,21].

Furthermore, other molecules are being studied on the angiogenic side, such as the hypoxia-inducible factor-2α (HIF-2α) inhibitor belzutifan [22,23].

With regard to ICI therapy, rechallenge with anti PD-1/PD-L1 therapy did not demonstrate significant benefits [24,25], therefore recent research has been seeking to explore new immune checkpoint targets.

In this review of the literature, we recollect the current knowledge on novel treatment strategies in the field of immunotherapy that are being investigated in RCC and analyze their mechanism of action, their activity and the clinical studies that are underway.

## 2. Methods

We performed a search on Pubmed/Medline, using the following keywords: “renal cell carcinoma” OR “kidney cancer” OR “RCC” AND “immunotherapy” OR “immune check-point inhibitors” OR “ICI” OR “CPI” OR “anti-PD1” OR “anti-PD-L1“. We also searched the words “TIGIT” OR “ILT4” OR “CAR-T” OR “LAG-3” OR “TIM-3” OR “vaccines” AND “renal cell carcinoma,” to address the biochemical nature of the novel targets and their role in the RCC treatment paradigm or its future perspectives. We selected pivotal registration studies and the most relevant studies in terms of how they were conducted, innovation, outcomes, statistical analysis and number of patients enrolled. We then performed a search on the Clinicaltrials.gov database for ongoing studies, both recruiting and not recruiting, using the keywords “renal cell carcinoma” AND “TIGIT” OR “ILT4” OR “CAR-T” OR “LAG-3” OR “TIM-3” OR “vaccines.” The search was carried out between 11 March and 31 May of the current year.

## 3. New Immune Pathways

Novel immune pathways are being studied as related to resistance to currently used ICIs and are being targeted with novel immune-related therapies. Herein, we discuss promising fields of research in the context of RCC.

### 3.1. T Cell Immunoglobulin and ITIM Domain (TIGIT)

T cell immunoglobulin and ITIM domain (TIGIT) is an inhibitory immunoglobulin receptor expressed on lymphocytes, consisting of an extracellular immunoglobulin variable domain, a type I transmembrane portion, and a short intracellular region with an inhibitory motif based on immunoreceptor tyrosine (ITIM) and an immunoglobulin tyrosine tail-like domain (ITT). First identified in 2009, TIGIT directly inhibits the proliferation of T cells and the action of natural killer (NK) cells [26,27].

TIGIT is a competitive ligand of the immune activator receptor CD226 or the DNAX-1 accessory molecule (DNAM-1) for CD155 and CD112 adhesion molecules, which are nectin receptors whose binding increases the activity of T and NK cells [28,29].

Jhonston et al. identified a highly specific 15-gene signature associated with tumor-infiltrating T cells with several co-inhibitors within TIGIT and programmed cell death-1 (PD-1) using gene expression data from the Cancer Genome Atlas collection of lung squamous carcinoma [30]. Similarly, this upregulation has been observed in ccRCC, colon, endometrial and breast cancers [31], for which several clinical trials are ongoing [32].

In particular, TIGIT has been shown to be mainly expressed in ccRCC tissue as compared with adjacent normal tissue [33].

### 3.2. Immunoglobulin-like Transcript 4 (ILT4)

Immunoglobulin-like transcript 4 (ILT4)—also named monocyte/macrophage immunoglobulin-like receptor 10 (MIR-10), lymphocyte immunoglobulin-like receptor (LIR) 2, or CD85d—is a type I transmembrane receptor with 4 extracellular tandem Ig-like domains, a transmembrane region of 23 amino acids and a cytoplasmic tail with 3 immunoreceptor tyrosine inhibitory motifs. Its main ligands are major histocompatibility complex (MHC) class I and human leukocyte antigens (HLAs) [34].

ILT4 belongs to a family of inhibitory and activating immunoglobulin-like transcripts which modulates activation of immune cells. It is predominantly expressed in innate immune cells, including macrophages, monocytes, granulocytes and dendritic cells. Furthermore, ILT4 is expressed in hematopoietic stem cells, osteoclast precursor cells, platelets and other neurons, being involved in their biological and functional regulation.

ILT4 has been shown to be overexpressed in malignant tumor cells from both hematopoietic and solid tumors and in the tumor stroma cell microenvironment, favoring tumor progression and metastasis [35].

Therefore, anti-ILT4 agents, in combination with other ICIs, could enhance the immune response against tumor cells. 

Siu et al. have demonstrated that a novel first-in-class human IgG4 monoclonal antibody targeting ILT4 (MK-4830), as monotherapy or in combination with pembrolizumab, triggered antitumor activity in patients with pretreated advanced solid tumors, including those whose disease had previously progressed during ICI while also maintaining a good safety profile [36].

### 3.3. Chimeric Antigen Receptor-T (CAR-T)

Chimeric antigen receptors (CARs) are chimeric receptor proteins designed to give T cells the ability to target a specific antigen. CAR-T is therefore a single receptor that combines both the antigen binding and T cell activation functions.

Thus, CAR-T cells are used in genetically modified immunotherapy, now widely used in the hematological field to treat some cancers, including diffuse large B-cell lymphoma, acute lymphoblastic leukemia and multiple myeloma. The standard approach is to collect T cells from patients, genetically alter them so they can recognize tumor cells, then infuse the resulting CAR-T cells back into patients (autologous transplant) or use those from a donor (allogeneic transplant).

Engineered CARs program T cells to recognize antigens expressed on cancer cells [37]. Therefore, the T cell linked to the antigen expressed on the tumor cell is active and exerts a cytotoxic action [38].

CD70 is highly expressed in RCC, making it a promising target for CAR-T cells.

Several phase I and phase II trials with CAR-T cells are ongoing for solid tumors, including RCC.

However, in solid tumors, unlike hematologic cancers, CAR-T cells have reduced efficacy due to their difficulty in penetrating the tumor. Moreover an immunosuppressive microenvironment restricts the efficacy [39].

Different toxicities are associated with CAR-T. Cytokine release syndrome (CRS) is one of the most important due to the secretion of multiple cytokines like IL-1, IL-6, TNF-α, and IL-10 that cause fever, arthralgia and myalgia. Similarly, endothelial activation driven by cytokines results in disruption of the blood–brain barrier (BBB), while a temporary leakage of cytokines into the cerebrospinal fluid and brain causes neurotoxicity [40]. Many other toxicities, like graft-versus-host disease, are observed in CAR-T therapy [41].

Chimeric antigen receptor natural killer (CAR-NK) is a new frontier of immunotherapy that promises to improve efficacy while reducing toxicity. Unlike T lymphocytes, NK cells have the ability to be transplanted into a new environment with different MHC expression patterns while maintaining their functionality and without triggering graft-versus-host disease or other toxicities, like CRS and neurotoxicity. With advancements in genetic modification technologies, NK cells can be further engineered, including the introduction of CARs and the knockout of inhibitory genes [42].

### 3.4. Lymphocytes Activation Gene 3 (LAG3)

Lymphocytes activation gene 3 (LAG3), also known as CD223, is an inhibitory receptor, a structural homolog of CD4, and is highly expressed on exhausted T cells and many other lymphocytic and non-lymphocytic cells. Its activity has been largely related to downregulating the immune response in both tumors and infections, but a thorough understanding of its ligands and its role in the immune pathways is still lacking [43].

Following the evidence that LAG3 tends to be overexpressed on exhausted T cells, the idea that T cell activity could be restored by inhibiting LAG3 has arisen, leading to anti-LAG3 immunotherapeutics. The association between anti-LAG3 relatlimab and anti-PD1 nivolumab in previously untreated advanced melanoma patients was studied in the randomized phase II/III RELATIVITY-047 trial, achieving a 12-month PFS rate of 47%, compared with 36% with nivolumab monotherapy [44]. Furthermore, the abovementioned association proved to be active and safe in heavily pretreated advanced melanoma patients who had progressed to a prior anti-PD(L)-1-containing regimen in the phase I/II RELATIVITY-020 trial [45].

In 2022, these results led the FDA to approve opdualag, an association between relatlimab and nivolumab, for the treatment of advanced melanoma [46].

Regarding RCC, an interesting study by Schoenfeld et al. investigated how LAG3 expression levels are, on average, lower at metastatic sites than those at primary RCC sites, and how this difference was more enhanced in patients with high-risk clinical features, such as those presenting with a larger primary tumor; with grade 4, IMDC poor-risk disease; or with brain metastases [47]. The authors further showed that higher LAG3 levels at metastatic sites may predict a greater response to immunotherapy and better survival outcomes after the development of metastatic disease.

The purpose of the open-label, randomized phase 2 FRACTION-RCC platform trial (NCT02996110) is to test the efficacy and safety of various combinations of nivolumab compared with nivolumab and ipilimumab in participants with advanced RCC that has progressed on or after ICI (participants undergoing anti-CTLA-4 therapy were eligible). This study uses an adaptive design to test different combination therapies: one arm consists of nivolumab and ipilimumab, another of nivolumab and relatlimab, another of nivolumab and BMS-986205, and the last consists of nivolumab and BMS-813160. This trial aims to recruit 200 patients with advanced RCC. The primary outcomes are ORR, PFS and duration of response (DOR). The results of the arm with patients treated with nivolumab plus ipilimumab were published in November 2022 and show that, with a median follow-up of 33.8 months, the ORR was 17.4% (entire population *n* = 46) with 8 patients achieving partial response and 19 patients achieving stable disease. The PFS rate at 24 weeks was 43.2%, mOS was 23.8 months and median DOR was 16.4 months.

### 3.5. T Cell Immunoglobulin and Mucin Domain 3 (TIM-3)

T cell immunoglobulin and mucin domain 3 (TIM-3) is a protein that is part of the TIM family and is identified by type 1 T helper cells (Th1) surface and then on other cell types such as type 17 T helper cells (Th17), monocytes, and macrophages. TIM-3 targets its ligand galectin-9 (Gal-9), inducing the depletion of Th1, resulting in peripheral immune tolerance and negative regulation of immune response. TIM-3 inhibition has been shown to be related to the blocking of tolerance induction in Th1 cells [48]. In addition, TIM-3 seems to be involved in the negative regulation of the production of IFN-Ɣ in CD8+ Tc1 cells [49]. TIM-3 overexpression has been observed in hepatocellular carcinoma (HCC) and appears to be related to poor prognosis, as the bond between TIM-3/Gal-9 promotes the depletion of T cells in HBV-related HCC [50]. A study by Yuan et al. assessed the prognostic role of TIM-3 overexpression ccRCC by analyzing 137 ccRCC tumor samples [51].

TIM-3 expression appears to be higher in tumoral tissue than in the adjacent normal renal tissue, and the short interfering RNA (siRNA)-mediated knockdown of TIM-3 inhibited proliferation and invasion in ccRCC cell lines. Furthermore, TIM-3 expression was found to be related to both cancer-specific survival and PFS and to be associated with poor prognosis.

### 3.6. Vaccines

Therapeutic cancer vaccines are based on the premise that a vaccine targets tumor-associated antigens through a cytotoxic immune response to these agents, inducing long-lasting immune responses by both establishing memory against tumor antigens and minimizing toxicity related to an off-target immune system. MHC molecules present peptide fragments derived from internal cellular proteins on the cell surface, thus allowing T cells to discriminate between healthy cells and diseased cells, including virus-infected and tumor cells [52].

Peptides that are predominantly present on tumor cells (and less so on healthy cells) are called tumor-associated peptides (TUMAPs). Vaccination with TUMAPs is believed to activate the immune system against cancer [53].

IMA901 is the first therapeutic vaccine developed for RCC. It is a vaccine consisting of ten selected TUMAPs that are naturally present in tumors.

IMA901 was evaluated in an early clinical trial with a single dose of cyclophosphamide (to deplete regulatory T cells), demonstrating that immune response to TUMAPs is associated with longer OS [54]. In contrast, the phase III IMPRINT trial, comparing sunitinib plus IMA901 versus sunitinib alone in first-line therapy for patients with metastatic ccRCC, showed no benefit in OS [55].

An alternative personalized vaccine approach is AGS-003. This is based on amplified tumor RNA, which was incorporated into autologous monocyte-derived dendritic cells (called rocapuldencel-T or AGS-003) and administered as a therapeutic vaccine. In a phase III trial, ASG-003 was combined with sunitinib versus sunitinib alone in intermediate or poor-risk metastatic ccRCC. No significant differences in PFS or OS were demonstrated [56].

The most recent approach targets tumor neoantigens. These are a specific class of tumor antigen and originate from somatic alteration. In many tumor types, neoantigen loads are correlated with response to ICI therapy [57]. In ccRCC, neoantigen load is lower than other immunotherapy sensitive cancer, like melanoma or lung cancer, but does not correlate with the response to ICI therapy [58].

Generating a personalized neoantigen vaccine is a feasible strategy that is achieved by sequencing a patient’s tumor genome and predicting which mutations generate peptides that might bind to specific HLA class I alleles.

### 3.7. Immunosuppressive Cells and Resistance

As already mentioned, immunosuppressive cells, such as TAM, MDSC, and Tregs, can contribute to a resistance to ICIs and have consequently become an important potential target with which to increase the efficacy of immunotherapy. TAMs have been thought to be involved in tumor development, proliferation and spread, with their high representation in TME appearing to be related to the poor prognosis of a broad spectrum of tumors. TAMs can undermine immune response by reducing T cells and NK activity, expressing proteins or releasing soluble factors, and by recruiting other immunosuppressive cells, such as Tregs [59]. Many studies have demonstrated the role of TAMs in immunotherapy resistance, thus the specific mechanism is still unknown [60]. MDSCs are a broad population of immature myeloid cells which massively increase during tumor development and proliferation. These cells both promote immune evasion, for example increasing PD-L1 expression to promote T cell anergy, and contribute directly to tumor proliferation via angiogenesis and by facilitating metastatization [61]. Lastly, Tregs, which present CD25 and CTLA-4 on their surface, play key roles in preventing autoimmune and inflammatory diseases. However, their increase in the TME suppresses anti-tumor immune response. Many preclinical studies have demonstrated that circulating Treg depletion can enhance anti-cancer immune response [62]. Immunosuppressive cells represent future potential targets for the development of new immunotherapeutics.

**Figure 1:**
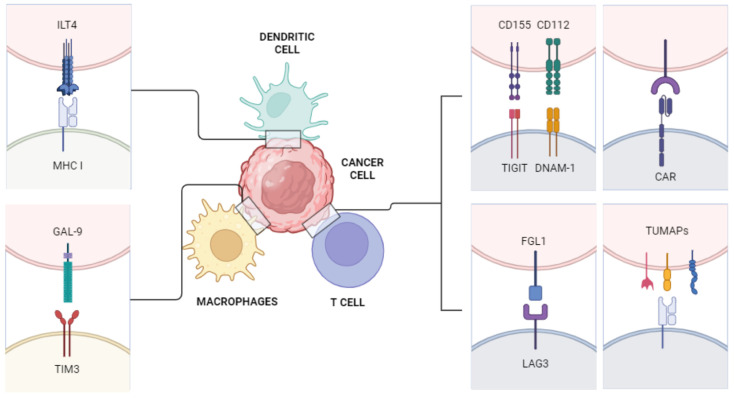
Novel immune pathways. ILT4: immunoglobulin-like transcript 4, which modulates activation of immune cells and appears to be overexpressed on the surface of solid and hematopoietic tumor cells; MHC I: major histocompatibility complex class I; GAL-9: galectin-9, a widely expressed receptor involved in immune response and tumor proliferation; TIM3: T cell immunoglobulin and mucin domain-containing protein 3; CD155: cluster of differentiation 115, often upregulated on tumor cells, contributing to proliferation; CD112: cluster of differentiation 112, a nectin receptor which, if activated, enhances the efficacy of T and NK cells; TIGIT: T cell immunoglobulin and ITIM domain, an immunoglobulin receptor expressed on lymphocytes, which directly inhibits the proliferation of T cells and the action of natural killer (NK) cells; DNAM-1: DNAX accessory molecule, one of the main NK cell-activating receptors; CAR: Chimeric antigen receptor; FGL1: fibrinogen-like protein 1; LAG3: lymphocyte activation gene-3, an inhibitory receptor which downregulates immune response in both tumors and infections; TUMAPs: multiple tumor-associated peptides.

The discussed immune pathways are depicted in Figure 1.

## 4. Trials Ongoing

### 4.1. Phase I–II Targeting TIGIT

Numerous studies on anti-TIGIT drugs are ongoing in RCC patients.

NCT05805501 is a randomized, open-label, three-arm phase II trial that evaluates the efficacy, safety, and pharmacokinetics of the anti-PD-1 and anti-LAG3 bispecific antibody tobemstomig (also known as RO7247669) in treatment-naive patients with unresectable or metastatic ccRCC. This trial is composed of two experimental arms—an arm A whose participants will receive the combination of tobemstomig and axitinib versus an arm B whose participants will receive the combination of tobemstomig, tiragolumab and axitinib—that are compared with a control arm (arm C) whose patients receive pembrolizumab plus axitinib.

NCT05259319 is a phase I trial that evaluates the safety and efficacy of the combination of the anti-PD-L1 atezolizumab with the anti-TIGIT tiragolumab—associated with concomitant or sequential stereotactic body radiation therapy (SBRT)—in patients with oligometastatic disease. In this study, one cohort consisted of patients progressing to first-line treatment.

Lastly, the NCT04626479 trial is a substudy of a larger research umbrella study (the phase Ib/II MK-3475-U03 umbrella trial), which aims to evaluate the safety and efficacy of experimental combinations of investigational agents (among which are favezelimab/pembrolizumab) in RCC. The substudy 03A (MK-3475-03A) involves participants with advanced untreated ccRCC (estimated *n* = 400), including a safety lead-in phase and an efficacy phase. On the other hand, the substudy 03B (MK-3475-03B) focuses on the second-line setting, enrolling pre-treated mRCC patients (estimated *n* = 370).

MK-3475-03A investigates the addition of vibostolimab (anti-TIGIT) to pembrolizumab in metastatic treatment-naive patients.

### 4.2. Phase I–II Targeting ILT4

The combination of pembrolizumab and MK-4830 is currently being studied in an umbrella trial that includes RCC (NCT04626518). CDX-585 is another anti-ILT-4 agent under investigation and is an open-label, non-randomized, multicenter, dose escalation and expansion study in patients with selected solid tumors, including RCC. It is currently open and enrolling (NCT05788484).

### 4.3. Phase I–II of CAR-T

Several phase I and II trials are ongoing to assess the safety and tolerability of CAR-T in patients with advanced RCC (NCT05420519, NCT06182735, NCT04696731, NCT04438083).

Carbonic anhydrase IX (CAIX), a genetic product downstream of the hyperactivation of the hypoxia inducible factor (HIF) pathway, is an interesting therapeutic target, being present with greater density in patients with ccRCC. NCT04969354 is a trial that evaluates the safety and efficacy of CAR-T cells targeting CAIX in metastatic RCC patients.

Furthermore, NCT03393936 is a dose escalation and dose expansion clinical study to evaluate the safety, tolerability and anti-tumor activity of the autologous CAR-T cells CCT 301-38 or CCT 301-59 in patients with mRCC. All of these studies are still recruiting and their results are awaited.

Another study investigated the administration of cells transduced with CAR VEGFR2, which inhibited the growth of tumor cells in different mouse strains (NCT01218867). This study is currently complete but final results have not yet been published.

For CAR-NK cells, NCT05703854 is a phase I/II study seeking to evaluate the safety, tolerability and optimal cell dose of CAR-NK in patients with advanced RCC.

### 4.4. Phase I–II Targeting LAG3

The DUET-4 trial (NCT03849469) is a phase I trial assessing the safety, pharmacokinetics and anti-tumor activity of the new anti-LAG3 drug XmAb22841, in monotherapy and in combination with pembrolizumab, in patients with advanced solid tumors, including RCC. This trial is complete, but results are yet to be reported.

One trial is currently studying the role of anti-LAG3 drugs in different settings of RCC. The STELLAR-002 trial (NCT05176483) aims to assess the activity of a new multi-targeted inhibitor of receptor tyrosine kinases, XL092, in combination with many immunotherapy agents, such as the anti-LAG3 relatlimab, in unresectable advanced solid tumors, including RCC. This trial is currently open and recruiting.

### 4.5. Phase I–II Targeting TIM-3

Two trials have tested two anti-TIM-3 drugs in advanced solid tumors, as follows: MBG453 (NCT02608268), also known as sabatolimab, and INCAGN02390 (NCT03652077). The first trial (NCT02608268) is a phase I/II trial built to assess the safety, pharmacokinetics and anti-tumor activity of the anti-TIM-3 drug MBG453 in advanced solid tumors, including RCC, administered in monotherapy or in combination with spartalizumab. The second study (NCT03652077) is a phase I trial which aims to investigate the safety and tolerability of INCAGN02390. While both of these studies are complete, their results have not yet been published.

### 4.6. Phase I–II of Vaccines

The NCT02950766 trial is currently assessing personalized neoantigen vaccines in combination with ipilimumab in ccRCC. Moreover, the NCT05269381 trial is investigating the safety and tolerability of tailored neoantigen vaccines in combination with pembrolizumab in advanced or metastatic malignancies, including RCC. Lastly, the addition of personalized neoantigen vaccines to the standard of care is being studied in the NCT05641545 trial.

We summarized ongoing phase I and phase II trials in Table 2.

### 4.7. Overview on Ongoing Trials

All of these new treatment combinations could represent a valid alternative to the current standard of care as first-line therapies and beyond in RCC, providing a broader inhibitory profile of multiple immune checkpoint targets. Thus, it has to be underlined that many of these studies involve small patient cohorts and often do not focus exclusively on RCC, needing further future large-scale studies to confirm efficacy and safety across different populations. Furthermore, despite the possible enhanced benefits in survival of targeting multiple immune checkpoints, there remains the relatively unknown potential of synergistics effects, the challenge of new resistance mechanisms and, as with every combination therapy, the possible increase in toxicity. For example, for second-line treatments and beyond, the use of anti-PD1 after progression on first-line ICIs may represent a challenge, considering the use of a similar target beyond progression. Lastly, and especially for CAR-T and vaccines, difficulties regarding manufacturing and costs may undermine the broad adoption of these treatment strategies in clinical practice.

There are currently no ongoing trials involving immunosuppressive cells such as Treg, MDSC, and TAM.

## 5. Conclusions

In both metastatic and adjuvant settings, immunotherapy is the cornerstone of treatment for RCC. However, an adaptive resistance from cancer cells may arise during ICI treatment. The mechanisms most frequently involved in the progressive resistance to ICI are the loss of response to IFN-gamma, the secretion of immunosuppressive cytokines, the loss of neoantigen expression, and the modifications of the tumor microenvironment related to the overexpression of TAM, MDSC, and Treg.

This has made it necessary to investigate new immune checkpoint inhibitor molecules that are able to overcome these crucial issues.

Drugs directed against TIGIT, ILT4, LAG-3, TIM-3 or therapeutic cancer vaccines are currently being studied in early-phase clinical trials, showing promising results. Additionally, trials are underway on the use of autologous and allogeneic transplants of CAR-engineered T cells and CAR-NK cells. These new immunotherapeutic targets could eventually change the therapeutic landscape of RCC, but the results of larger studies are needed.

## Figures and Tables

**Table 1:** Outcomes of first-line immune-based combinations trials.

Trial	Treatment	Median Follow Up, Months	OS HR	mOS, Months	PFS HR	mPFS, Months	ORR, %	Reference
Checkmate 214	Nivolumab + ipilimumab (*n* = 550) vs. sunitinib (*n* = 546)	96	0.72	52.7 vs. 37.8	0.88	12.4 vs. 12.3	39 vs. 32 CR 12%	Motzer RJ et al., N Engl J Med. 2018 [8]
Keynote-426	Pembrolizumab + axitinib (*n* = 432) vs. sunitinib (*n* = 429)	67	0.84	47.2 vs. 40.8	0.69	15.7 vs. 11.1	61 vs. 40 CR 2%	Rini BI et al., N Engl J Med. 2019 [9]
Checkmate-9ER	Nivolumab + cabozantinib (*n* = 323) vs. sunitinib (*n* = 328)	55	0.77	46.5 vs. 36.0	0.58	16.4 vs. 8.4	56 vs. 28 CR 13.6%	Choueiri TK et al., N Engl J Med. 2021 [10]
Clear	Pembrolizumab + lenvatinib (*n* = 355) vs. sunitinib (*n* = 357)	48	0.79	53.7 vs. 54.3	0.47	23.9 vs. 9.2	71 vs. 37 CR 18%	Motzer RJ et al., N Engl J Med. 2021 [11]

OS: overall survival; HR: hazard ratio; mOS: median overall survival; PFS: progression-free survival; mPFS: median progression-free survival; ORR: objective response rate; CR: complete response.

**Table 2:** Ongoing phase I and phase II trials in metastatic RCC patients.

ClinicalTrials.gov ID	Phase	Setting	Drug	Primary Endpoints and Phase	Estimated Primary Completion Date
NCT05805501	II	Untreated, unresectable locally advanced or metastatic RCC	Tobemstomig (RO7247669) plus axitinib with or without tiragolumab versus pembrolizumab plus axitinib	Efficacy, safety, and pharmacokinetics	September 2024
NCT05259319	I	Second-line therapy after an anti-angiogenic plus immunotherapy or immunotherapy alone	Atezolizumab and tiragolumab, with concomitant or sequential stereotactic body radiation therapy	Safety and efficacy	December 2024
NCT04626479	Ib–II	First-line in untreated patient with advanced or metastatic RCC	Vibostolimab/ pembrolizumab	Safety and efficacy	May 2026
NCT05788484	I	Relapsed, locally advanced or metastatic setting after standard treatment	CDX-585	Dose escalation	December 2024
NCT04626518 Substudy 03B MK-3475-03B	Ib–II	Second and later lines	Pembrolizumab + MK-4830	Safety and efficacy	September 2025
NCT05420519	I	Advanced or metastatic RCC	CD70 CAR-T cells	Safety and tolerability	December 2024
NCT04969354	I	Advanced or metastatic RCC	CAIX-targeted CAR-T Cells	Safety and efficacy	September 2026
NCT03393936	I–II	Advanced or metastatic RCC	CCT301-38 CCT301-59 CART-T Cells	Safety, tolerability and anti-tumor activity	June 2023
NCT06182735	I	Advanced or metastatic RCC	Cyclophosphamide plus fludarabine plus infusion of CAR-NKT Cells	Safety, tolerability, PK, and preliminary efficacy	January 2025
NCT04696731	I	Advanced or metastatic RCC	Cyclophosphamide, fludarabine, ALLO-647, ALLO-316	Safety and efficacy	August 2025
NCT04438083	I	Advanced, relapsed or refractory RCC	CTX130	Safety and efficacy	February 2027
NCT05176483	Ib	Advanced or metastatic RCC	XL092, novolumab, ipilimumab, relatlimab	Safety, tolerability, PK, preliminary antitumor activity, and effect	February 2026
NCT05641545	Ib	Advanced or metastatic RCC	Personalized neoantigen vaccine plus standard of care.	Safety and clinical toxicity	December 2024
NCT05269381	I	Advanced or metastatic solid tumors (including RCC)	Cyclophosphamide, neoantigen peptide vaccine, pembrolizumab, sargramostim	Safety and tolerability	February 2025
NCT05703854	I–II	Advanced or metastatic solid tumors (including RCC)	CAR.70/IL15-transduced CB-derived NK cells, fludarabine phosphate, cyclophosphamide	Safety, tolerability, and optimal cell dose	September 2025

RCC: renal cell carcinoma.
